# Post-infectious and post-acute sequelae of critically ill adults with COVID-19

**DOI:** 10.1371/journal.pone.0252763

**Published:** 2021-06-17

**Authors:** Halah Ibrahim, Syed Athar, Thana Harhara, Shahad Abasaeed Elhag, Salma MElnour, Hoor H. Sukkar, Ashraf M. Kamour

**Affiliations:** 1 Department of Medicine, Sheikh Khalifa Medical City, Abu Dhabi, United Arab Emirates; 2 Education Institute, Sheikh Khalifa Medical City, Abu Dhabi, United Arab Emirates; San Giuseppe Hospital, ITALY

## Abstract

**Background:**

Data on the post-acute and post-infectious complications of patients who have recovered from severe coronavirus disease 2019 (COVID-19) are limited. While studies report that approximately 5–15% of COVID-19 hospitalized patients require intensive care and mechanical ventilation, a substantially higher number need non-invasive ventilation and are subject to prolonged hospitalizations, with long periods of immobility and isolation. The purpose of this study is to describe the post-infectious sequelae of severe viral illness and the post-acute complications of intensive care treatments in critically ill patients who have recovered from severe COVID-19 infection.

**Methods:**

We performed a retrospective chart review of adult patients initially hospitalized with confirmed COVID-19 infection, who recovered and were transferred to a general medical ward or discharged home between March 15, 2020 and May 15, 2020, dates inclusive, after an intensive care unit (ICU) or high dependency unit (HDU) admission in a designated COVID-19 hospital in the United Arab Emirates. Demographic data, underlying comorbidities, treatment, complications, and outcomes were collected. Descriptive statistical analyses were performed.

**Results:**

Of 71 patients transferred out of ICU (n = 38, 54%) and HDU (n = 33, 46%), mean age was 48 years (SD, 9.95); 96% men; 54% under age 50. Mean ICU stay was 12.4 days (SD, 5.29), HDU stay was 13.4 days (SD, 4.53). Pre-existing conditions were not significantly associated with developing post-acute complications (Odds Ratio [OR] 1.1, 95% confidence interval [CI] 0.41, 2.93, p = 1.00). Fifty nine percent of patients had complications; myopathy, swallowing impairments, and pressure ulcers were most common. Delirium and confusion were diagnosed in 18% (n = 13); all were admitted to the ICU and required mechanical ventilation. Of note, of all patients studied, 59.2% (n = 42/71) had at least 1 complication, 32.4% (n = 23) had at least 2 complications, and 19.7% (n = 14) suffered 3 or more sequelae. Complications were significantly more common in ICU patients (n = 33/38, 87%), compared to HDU patients (n = 9/33, 27%) (OR 17.6, 95% CI 5.23, 59.21, p <0.05).

**Conclusion:**

In a subset of critically ill patients who recovered from severe COVID-19 infection, there was considerable short-term post-infectious and post-acute disability. Long-term follow-up of COVID-19 survivors is warranted.

## Introduction

The coronavirus disease 2019 (COVID-19) pandemic has disrupted healthcare systems worldwide. To increase critical care capacity, hospitals have suspended routine services and redeployed healthcare staff to accommodate the large surges of critically ill patients. While studies report that approximately 5–15% of COVID-19 hospitalized patients require intensive care and mechanical ventilation [1, 2], a substantially higher number need supplemental oxygen or non-invasive ventilation and are subject to prolonged hospitalizations, with long periods of immobility and isolation. As hospitals continue to manage acute patients, projections suggest a subsequent surge in post-acute care demand [3]. This case series describes the post-infectious sequelae of severe viral illness and the post-acute complications of intensive care treatments in critically ill patients who have recovered from severe acute respiratory syndrome coronavirus 2 (SARS-CoV-2) infection.

## Methods

This retrospective study was performed at Sheikh Khalifa Medical City (SKMC), a large government hospital in the United Arab Emirates (UAE) that became a designated COVID-19 treatment center. Study participants included all patients hospitalized with SARS-CoV-2 infection confirmed by positive polymerase chain reaction (PCR) testing of nasopharyngeal specimens, who were transferred to a general medical ward or discharged home between March 15, 2020 and May 15, 2020, dates inclusive, after an intensive care unit (ICU) or high dependency unit (HDU) admission. Critical care patients in this study include all HDU and ICU patients. HDU patients required non-invasive ventilation or high flow oxygen, or inotropic support as needed, whereas patients with respiratory failure requiring mechanical ventilation were admitted to the ICU. Clinical outcomes were monitored until May 30, 2020, the final date of follow-up.

From May 15^th^ through June 30^th^ 2020, using a standardized data collection form, three trained physicians reviewed patient electronic medical records, nursing notes and laboratory results and collected data on age, gender, comorbidities, highest level of respiratory support (mechanical ventilation, non-invasive ventilation, oxygen mask), results of subsequent PCR tests and outcomes (including length of stay, abnormal liver, cardiac and kidney function tests or kidney replacement therapy, neurologic or psychiatric impairments and muscle weakness or mobility impairment). Race and ethnicity data were collected from UAE national identification cards located in the electronic medical record. Acute kidney injury was identified as an increase in serum creatinine by ≥0.3mg/dL (≥26.5 mol/L) within 48 hours or an increase in serum creatinine to ≥1.5 times baseline within the prior 7 days compared with the preceding 1 year of data in acute care medical records, based on the Kidney Disease: Improving Global Outcomes (KDIGO) definition [4]. Acute hepatic injury was defined as an elevation in aspartate aminotransferase or alanine aminotransferase of >15 times the upper limit of normal. Acute cardiac injury was characterized by the presence of serum levels of high-sensitivity cardiac troponin I above the 99th percentile upper reference limit. Myopathy was diagnosed in patients with muscle weakness and bedside manual testing of muscle strength revealed muscle atrophy, flaccid limb weakness or decreased deep tendon reflexes [5]. Delirium was diagnosed based on clinical exam by treating physicians in patients with acute confusional state or altered or fluctuating mental status [6].

Patients were prescribed multiple treatments during their acute hospitalization. As per UAE Department of Health guidelines, after discussion with patients and informed consent provided, all hospitalized COVID-19 patients received hydroxychloroquine and lopinavir-ritonavir or favipiravir. Unless there was a contraindication to anticoagulation, every hospitalized patient was placed on prophylactic anticoagulation, and therapeutic enoxaparin was prescribed for all patients with a D-dimer above 3 μg/ml, with early recognition and treatment of venous thromboembolism. Critically ill patients with elevated interleukin 6 levels greater than 100 pg/ml or a 5-fold increase from prior level received tocilizumab; and some patients were enrolled in a convalescent plasma clinical trial. Steroids and remdesivir were not prescribed to our COVID-19 patients during the time of this study.

Patients were discharged home when they met World Health Organization’s initial criteria for discontinuation of quarantine, namely after documentation of absence of fever and respiratory symptoms for 3 consecutive days and 2 negative SARS-CoV-2 PCR tests at least 24 hours apart [7]. Of note, current WHO criteria no longer require re-testing [7]. Additional discharge requirements included the ability to independently perform activities of daily living (ADLs) or having assistance to support with ADLs.

Descriptive statistical analyses were performed to calculate the proportions of patients developing complications. Odds ratios were calculated employing Chi-square test with Yates’ correction to derive p values. This study follows the Strengthening the Reporting of Observational Studies in Epidemiology (STROBE) reporting guideline [8]. The Abu Dhabi Health COVID-19 Research Ethics Committee approved the project with a waiver of informed consent.

## Results

During the study period, 71 patients with confirmed COVID-19 infection were transferred out of the ICU (n = 38, 54%) and HDU (n = 33, 46%) to the general medical ward. Table 1 lists patient demographic characteristics. The mean age was 48 years (SD, 9.95); 96% were men. Mean ICU stay was 12.4 days (SD, 5.29), and HDU stay was 13.4 days (SD, 4.53). Of patients admitted to ICU, 87% (n = 33) required mechanical ventilation, for an average of 11.3 days (SD, 5.29). Approximately 39% of the patients did not have known comorbidities prior to hospitalization. Of patients with pre-existing conditions, 49% had diabetes mellitus, and 31% had hypertension. The presence of pre-existing conditions was not significantly associated with developing post-acute complications (Odds Ratio [OR] 1.1, 95% confidence interval [CI] 0.41, 2.93, p = 1.00). Of note, 54% of patients admitted to critical care were under the age of 50. Of patients who developed complications, 57% were less than 50 years old. Length of stay was also not associated with developing complications (OR 0.68, 95% CI 0.26, 1.78, p = 0.80). Except for a higher percentage of male patients, demographics of this cohort did not differ substantially from the total critical care COVID-19 patient population (Table 2). Demographic differences between this survivor cohort and critical care patients who died of COVID-19 infection are listed in Table 3, and are only significant for older age and increased prevalence of comorbidities amongst the fatalities.

**Table 1:** Characteristics of adult patients transferred from critical care units to general medical ward.

	No. (%)		
	All adults (N = 71)	HDU admission (n = 33)	ICU admission (n = 38)
**Age, mean, years**			
**<50**	38 (54%)	15 (45.5%)	23 (60.5%)
**≥50**	33 (46%)	18 (54.5%)	15 (39.5%)
**Sex**			
**Male**	68 (95.8%)	32 (97.0%)	36 (94.7%)
**Female**	3 (4.2%)	1 (3.0%)	2 (5.3%)
**Ethnicity**^a^			
**Arab**	6 (8.4%)	4 (12.1%)	2 (5.3%)
**African**	2 (2.8%)	0 (0%)	2 (5.3%)
**South Asian**	54 (76.1%)	23(69.7%)	31 (81.6%)
**Southeast Asian**	9 (12.7%)	6 (18.2%)	3 (7.9%)
**Comorbidities**^b^			
**No comorbidities**	28 (39.4%)	12 (36.4%)	16 (42.1%)
**Asthma or COPD**	4 (5.6%)	1 (3.0%)	3 (7.9%)
**Hypertension**	14 (19.7%)	8 (24.2%)	6 (15.8%)
**Diabetes**	22 (31.0%)	11 (33.3%)	11 (28.9%)
**Chronic Kidney Disease**	1 (1.4%)	1 (3.0%)	0 (0%)
**Immunosuppression**^c^	2 (2.8%)	1 (3.0%)	1 (2.6%)
**Malignancy**^d^	2 (2.8%)	1 (3.0%)	1 (2.6%)
**Obesity (BMI ≥30)**^e^	18 (25.4%)	11 (33.3%)	7 (18.4%)
**Highest level of respiratory support**			
**Nasal cannula/face mask**	6 (8.4%)	6 (18.2%)	0 (0%)
**High-flow oxygen**	17 (23.9%)	15 (45.5%)	2 (5.3%)
**Noninvasive ventilation**	15 (21.1%)	12 (36.4%)	3 (7.9%)
**Invasive/mechanical ventilation**	33 (46.8%)	0 (0%)	33 (86.8%)

Abbreviations: HDU, high dependency unit; ICU, intensive care unit; COPD, chronic obstructive pulmonary disease; BMI, body mass index

^a^Race and ethnicity data were collected from UAE national identification cards located in the electronic medical record.

^b^Comorbidities listed here are defined as medical diagnoses included in medical history by ICD-10 coding.

^c^Immunosuppression include HIV, history solid organ transplant or autoimmune disease.

^d^Malignancy includes active solid organ or hematologic malignancy (not in remission) or receiving active chemotherapy.

^e^Obesity was defined as BMI ≥ 30. Body mass index is calculated as weight in kilograms divided by height in meters squared.

**Table 2:** Demographics of patients transferred from critical care units to general medical ward and all COVID-19 critical care patients.

	No. (%)		
	Discharged (N = 71)	All ICU/ HDU admissions (N = 669)	p Value
**Age, mean, years**			
**<50**	38 (54%)	368 (55%)	0.90
**≥50**	33 (46%)	301 (45%)	0.90
**Sex**			
**Male**	68 (95.8%)	589 (88.0%)	**<0.05**
**Female**	3 (4.2%)	80 (12.0%)	**<0.05**
**Comorbidities**^a^			
**No comorbidities**	28 (39.4%)	320 (47.8%)	0.21
**Asthma or COPD**	4 (5.6%)	6 (0.8%)	**<0.05**
**Hypertension**	14 (19.7%)	211 (30.2%)	**<0.05**
**Diabetes**	22 (31.0%)	230 (34.4%)	0.60
**Chronic Kidney Disease**	1 (1.4%)	22 (3.3%)	0.71
**Immunosuppression**^b^	2 (2.8%)	10 (1.5%)	0.32
**Malignancy**^c^	2 (2.8%)	10 (1.5%)	0.32
**Obesity (BMI ≥30)**^d^	18 (25.4%)	170 (25.4%)	1.00

Abbreviations: HDU, high dependency unit; ICU, intensive care unit; COPD, chronic obstructive pulmonary disease; BMI, body mass index

^a^Comorbidities listed here are defined as medical diagnoses included in medical history by ICD-10 coding.

^b^Immunosuppression include HIV, history solid organ transplant or autoimmune disease.

^c^Malignancy includes active solid organ or hematologic malignancy (not in remission) or receiving active chemotherapy.

^d^Obesity was defined as BMI ≥ 30. Body mass index is calculated as weight in kilograms divided by height in meters squared.

**Table 3:** Comparison of characteristics of COVID-19 survivors and deaths.

	No. (%)		
	Survivor Cohort (N = 71)	Deaths (N = 41)	p Value
**Age, mean, years**			**<0.02**
**<50**	38 (54%)	12 (29.3%)	
**≥50**	33 (46%)	29 (70.7%)	
**Sex**			0.26
**Male**	68 (95.8%)	37 (90.2%)	
**Female**	3 (4.2%)	4 (9.8%)	
**Comorbidities**^a^			
**No comorbidities**	28 (39.4%)	6 (14.6%)	**<0.05**
**Asthma or COPD**	4 (5.6%)	3 (7.3%)	0.71
**Hypertension**	14 (19.7%)	14 (34.1%)	0.11
**Diabetes**	22 (31.0%)	14 (34.1%)	0.83
**Chronic Kidney Disease**	1 (1.4%)	1 (2.4%)	1.00
**Immunosuppression**^b^	2 (2.8%)	0 (0%)	0.53
**Malignancy**^c^	2 (2.8%)	1 (2.4%)	1.00
**Obesity (BMI ≥30)**^d^	18 (25.4%)	12 (29.3%)	0.66

Abbreviations: HDU, high dependency unit; ICU, intensive care unit; COPD, chronic obstructive pulmonary disease; BMI, body mass index

^a^Comorbidities listed here are defined as medical diagnoses included in medical history by ICD-10 coding.

^b^Immunosuppression include HIV, history solid organ transplant or autoimmune disease.

^c^Malignancy includes active solid organ or hematologic malignancy (not in remission) or receiving active chemotherapy.

^d^Obesity was defined as BMI ≥ 30. Body mass index is calculated as weight in kilograms divided by height in meters squared.

The primary outcomes are summarized in Table 4. Forty one percent had no documented sequelae. Of note, of all patients studied, 59.2% (n = 42/71) had at least 1 complication, 32.4% (n = 23) had at least 2 complications, and 19.7% (n = 14) suffered 3 or more sequelae. Post-infectious and post-acute complications were significantly more common in ICU patients (n = 33/38, 87%), as compared to those admitted to HDU (n = 9/33, 27%) (OR 17.6, 95% CI 5.23, 59.21, p <0.05). Myopathy, swallowing problems, and pressure ulcers were the most common post-acute complications, each affecting approximately one third (n = 22, 30%) of patients. Seventy-one percent of these patients had both swallowing difficulties and pressure ulcers. The vast majority of myopathy occurred in ICU patients (n = 21), as compared to a single case in the HDU (OR 39.53 95% CI 4.89, 319.78, p <0.0001). Patients who developed myopathy had an average length of ICU stay of 14.5 days (SD, 4.45), compared to 8.8 days (SD, 3.39) for those who did not have this complication. Delirium and confusion were diagnosed in 18% (n = 13) of patients, all of whom were admitted to the ICU and required mechanical ventilation. Four patients (5%) received psychiatric consultations- 1 for depression and 3 for anxiety.

**Table 4:** Post-infectious and post-acute complications of COVID-19 patients transferred out of critical care units.

Clinical Outcome	No. (%)		
	All adults (N = 71)	HDU admission (n = 33)	ICU admission (n = 38)
**Length of stay**^a^**, mean, days (SD)**	12.4 (5.29)	13.4 (4.53)	12.3 (5.29)
**Renal**	68 (95.8%)		
Electrolytes Imbalance	49 (69.0%)	14 (42.4%)	35 (92.1%)
Acute Kidney Injury^b^	11 (15.5%)	2 (6.1%)	9 (23.7%)
Renal Replacement Therapy	8 (11.3%)	0 (0.0%)	8 (21.1%)
**Hepatic**			
Acute Hepatic Injury^c^	11 (15.5%)	6 (18.2%)	5 (13.2%)
**Cardiac**			
Acute Cardiac Injury^d^	1 (1.4%)	0 (0.0%)	1 (2.6%)
**Pulmonary**			
Post-acute supplemental oxygen	48 (67.6%)	22 (66.7%)	26 (68.4%)
**Hematologic**			
VTE	1 (1.4%)	0 (0.0%)	1 (2.6%)
**Musculoskeletal**	44 (62.0%)		
Myopathy^e^/muscle weakness	22 (31.0%)	1 (3.0%)	21 (55.3%)
Pressure ulcer/wound	22 (31.0%)	1 (3.0%)	21 (55.3%)
**Neurologic**	39 (54.9%)		
Delirium/confusion^f^	13 (18.3%)	0 (0.0%)	13 (34.2%)
Ataxia/gait imbalance	4 (5.6%)	0 (0.0%)	4 (10.5%)
Swallowing impairment	22 (31.0%)	1 (3.0%)	21 (55.3%)

Abbreviations: HDU, high dependency unit; ICU, intensive care unit; SD, standard deviation; VTE, venous thromboembolism

^a^Length of stay begins with admission time and ends with discharge time, days

^b^Acute kidney injury was identified as an increase in serum creatinine by ≥0.3mg/dL (≥26.5 mol/L) within 48 hours or an increase in serum creatinine to ≥1.5 times baseline within the prior 7 days compared with the preceding 1 year of data in acute care medical records. Acute kidney injury is calculated only for patients with record of baseline kidney function data available and without a diagnosis of end-stage kidney disease.

^c^Acute hepatic injury was defined as an elevation in aspartate aminotransferase or alanine aminotransferase of >15 times the upper limit of normal.

^d^Acute cardiac injury was characterized by the presence of serum levels of high-sensitivity cardiac troponin I above the 99th percentile upper reference limit.

^e^Myopathy was diagnosed when bedside manual testing of muscle strength revealed muscle atrophy, flaccid limb weakness or decreased deep tendon reflexes.

^f^Delirium was diagnosed based on clinical exam by treating physicians in patients with acute confusional state or altered or fluctuating mental status.

Eleven patients (16%) developed renal impairment, 9 in ICU and 2 in HDU (OR 4.81 95% CI 0.96, 24.15, p = 0.05). Most patients who developed renal impairment required renal replacement therapy (n = 8; 72%). On average, patients spent 17.5 days receiving hemodialysis. None of the patients required renal replacement therapy on discharge. Acute liver injury developed in 16% (n = 11) of critical care patients and was transient in all cases. Acute cardiac injury was diagnosed in 1 patient, and only 1 patient was diagnosed with a thrombotic complication. There were no major adverse bleeding events in this patient cohort. Specifically, none of the patients were diagnosed with an intracranial hemorrhage or gastrointestinal bleed that required blood transfusion or endoscopic intervention. Following discharge from critical care, 46% of patients (n = 48) required supplementary oxygen (95% CI 0.56, 0.77) for an average of 5.9 days (SD, 3.53). None of the patients were discharged home on oxygen. As of May 30, 2020, 14% (n = 10) of the cohort was still hospitalized.

## Discussion

High death rates and substantial disability have been reported in COVID-19 patients admitted to critical care, particularly those requiring mechanical ventilation [9]. Our findings show that in a subset of critically ill patients who recovered and were transferred to the general medical ward or discharged home, there was considerable short-term post-infectious and post-acute disability. Studies have documented that patients with underlying medical comorbidities were more likely to suffer severe complications from SARS-CoV-2 [10]. In this cohort, complications were not associated with prior history of medical comorbidities. Also, most complications occurred in patients under age 50. The large numbers of South Asian patients and the majority male population in our study reflects the demographics of the COVID-19 hospitalized patients in the UAE, which included a large number of laborers, a primarily male population from South and Southeast Asia. The preponderance of critically ill male patients is also consistent with other studies, which document more severe disease in men [9, 10].

COVID-19 can cause severe pulmonary symptoms and respiratory failure. Although 68% of the patients studied required supplemental oxygen on the general medical ward, none of the patients were discharged home on oxygen therapy. It is unknown, however, how many continued to have dyspnea or decreased exercise capacity post-discharge. The rates of organ injury in our patient population were consistent with other studies that have documented organ injury in hospitalized COVID-19 patients. Systematic reviews and meta-analyses have documented acute kidney injury in 28% of infected patients, with 9% requiring kidney replacement therapy, and hepatic dysfunction has been reported in 14–53% of patients with COVID-19 [11, 12].

Our findings are comparable to studies of non-COVID-19 critically ill patients with sepsis, which have also documented high rates of organ damage. For example, a prospective multinational European study of over 1100 patients with sepsis across 198 ICUs noted a 39% incidence of severe acute kidney injury, with 13.5% requiring renal replacement therapy [13]. Similarly, 11.3% of patients in this study required renal replacement therapy. Also, the finding of hepatic injury in 15% of the study population is comparable to a large multicenter Austrian study, in which acute hepatic dysfunction was found in approximately 10% of critically ill patients, many of whom were septic [14]. Further, a study of ICU patients with non-COVID-19 acute respiratory distress syndrome documented myopathy and weakness at hospital discharge in 36% of the patients [15], similar to the 31% incidence in this patient cohort. Finally, approximately 18% of patients in our study developed delirium. Research shows that the prevalence of delirium in ICU patients can be as high as 50%, but without explicit and objective delirium monitoring, up to 75% of delirium diagnoses are missed [6, 16]. A case series of over 200 patients with COVID-19 hospitalized in Wuhan, China reported neurologic symptoms in 36.4%, but only 7.5% had any chart documentation of delirium [17]. As there was no specific screening for mild cognitive impairments or symptoms of anxiety or depression in our patients, neurologic and psychiatric complications are likely grossly under-reported. To date, it is unclear if CNS manifestations of SARS-CoV-2 infection are caused by the virus directly or sequelae of critical illness. In our study, all of the patients who developed delirium were admitted to the ICU. This is consistent with other studies that report increasing prevalence with disease severity [16], but may also suggest that delirium is associated more with critical illness and ICU stay, rather than the infection itself. In a recent meta-analysis, delirium was significantly associated with long-term cognitive decline [18]. As such, despite the heavy clinical workload brought on by the pandemic, implementation of screening methods using validated instruments, such as Confusion Assessment Method (CAM), and mitigation strategies for delirium in critically ill COVID-19 patients is of utmost importance [6]. We are concerned that only 4 patients received psychiatric consultation on the medical ward. Studies of patients hospitalized during the SARS outbreak revealed significant short-term and long-term psychological distress [19]. Perceived social support during their hospitalization helped to mitigate psychological distress and promoted patient mental health and wellbeing [20]. During COVID-19 pandemic restrictions, healthcare staff should make every effort to provide regular updates to patients and use virtual technology to facilitate communication between patients and their families.

Understanding of the myriad and complex post-infectious and post-acute sequelae of COVID-19 infection is still evolving. Early identification of clinicopathological signs may better inform treatment plans and lead to improved clinical results. The use of standardized frameworks, such as the ABCDEF bundle, can provide a holistic approach that can help optimize critical care patient recovery and outcomes [21]. It is reassuring that most of the short-term post-infectious disablement in this cohort improved over time and was modifiable with rehabilitation interventions, with the vast majority of patients discharged home. However, it is unclear how many patients ultimately returned to their pre-infection functional status. As such, the effects of prolonged critical care hospitalizations for COVID-19 can place heavy burdens on patients, their caregivers, and their communities.

Further, studies in patients admitted with stroke or chronic lung disease have confirmed that early rehabilitation improves clinical and functional outcomes and is significantly associated with a lower risk of mortality at 1 year [22]. The same is likely true for COVID-19 survivors. Yet, current infection control measures may impair post-acute care services and delay clinical and functional recovery, particularly for patients with delirium who continue to face restricted visitation and decreased physical contact from healthcare staff. To minimize long-term complications of COVID-19 infection, hospitals and post-acute care facilities need to ramp up capacity and capability to manage both COVID-19 positive and post-infectious patients. Further, individualized rehabilitation programs are necessary to focus on disabilities specific to each patient. For example, the high rates of complications supports the long-term clinical follow up of survivors after discharge, with a focus on physical therapy, wound care, and neurological sequelae.

It is not surprising that the majority of complications were seen in ICU patients, particularly those who were mechanically ventilated. These patients also likely had more severe disease. Thus, it remains unclear if the complications are direct manifestations of viral infection or post-ICU sequelae. Our findings suggest that critical care physicians should optimize the use of high flow oxygen non-invasive ventilation and focus clinical efforts into avoiding intubation and mechanical ventilation in hypoxemic COVID-19 patients whenever feasible. Recent studies support this strategy [23]. Further, numerous different medications, including hydroxychloroquine and tocilizumab, were used to treat this severely ill patient population. Given the toxicity profiles and now documented adverse medication side effects [24], these treatments may have potentially contributed to some of the complications seen. Recent studies have shown improved clinical outcomes in COVID-19 patients treated with remdesivir or with steroids [25]. It is possible that newer treatment protocols will result in decreased rates of complications in COVID-19 survivors. The low incidence of thromboembolic complications in our patient cohort is notable, and may be due to the early use of prophylactic anticoagulation in all hospitalized COVID-19 patients and awareness and early treatment of the thrombotic complications of COVID-19 infection. Although there were no major bleeding complications in this patient cohort, recent guidelines explicitly recommend against the use of high dose anticoagulation in critically ill patients with COVID-19 infection, and our institution protocols have been modified accordingly [26]. Further studies are needed to identify the optimal anticoagulation protocol in critically ill patients with hypoxemic respiratory failure.

Diabetes is the most common comorbidity in this critically ill patient cohort, as well as the most common pre-existing condition in all COVID-19 patients admitted to our institution. Although the presence of pre-existing conditions was not significantly associated with developing post-acute complications in our study, studies confirm that diabetes is a risk factor for COVID-19 infection and contributes to illness severity and mortality [27]. Obesity is also considered an independent risk factor for increased morbidity and mortality in COVID-19 [6]. In fact, studies have documented that obesity increases all cause morbidity in the general population [28]. Studies of the effect of obesity on mortality in hospitalized ICU patients have had mixed results, however, with some showing a longer length of hospitalization but a protective effect on mortality in mechanically ventilated patients [29]. In our critical care population, the presence of obesity did not correlate with morbidity or mortality. Further studies are necessary to determine the specific effects of obesity on critically ill hospitalized patients, both with and without COVID-19 infection.

The inclusion of HDU patients in our analysis is important because the current literature is limited regarding short-term and midterm complications for hospitalized patients with severe COVID-19 illness who require intensive care but not mechanical ventilation. Study limitations include limited generalizability given the retrospective design at a single center, with a small sample size and predominantly male patient population. As such, with a study population homogeneous in sex, ethnicity and treatment center, the rates of complications could differ substantially in a different population. The single center limitation is partially offset by the fact that the hospital served as a designated COVID-19 referral center and treated critically ill patients from throughout the country. Also, myopathy was diagnosed by physical exam and not confirmed by neurophysiological studies. Further, without standardized screening tools for delirium, neurocognitive and psychiatric impairments and physical impairments, such as mild weakness or disequilibrium, it is likely that many disabilities are underestimated. Although the same multidisciplinary team of critical care physicians, nurses and physiotherapists cross-covered ICU and HDU patients during the pandemic, it is possible that recognition of the risk and importance of some complications, such as myopathy and delirium, in mechanically ventilated patients by clinical staff in the ICU might have exaggerated the difference in higher prevalence of these conditions in the ICU patients, as compared with the non-intubated HDU patients. Finally, the study focused on short-term complications in critically ill COVID-19 patients and did not assess all possible complications of COVID-19 infection.

## Conclusion

Our study of short-term sequelae reveals a high rate of post-infectious and post-acute complications in COVID-19 survivors, irrespective of age and prior comorbidities. Using high flow oxygen and non-invasive ventilation to delay intubation and mechanical ventilation, minimizing ICU length of stay, screening and mitigation of common critical illness complications, including pressure ulcers, myopathy and delirium, and early initiation of physiotherapy are important strategies to minimize the rate and severity of complications. Early identification and management of post- COVID-19 sequelae are important to improve functioning and promote physical and psychological recovery, as well as social re-integration. Longitudinal follow up of COVID-19 survivors is necessary to monitor long-term outcomes and understand the natural history of the disease. The medical and economic impact of COVID-19 on global healthcare systems will likely be felt for months and years to come.

## Supporting information

S1 Table Characteristics of adult patients transferred from critical care units to general medical ward. (PDF)

S2 Table Demographics of patients transferred from critical care units to general medical ward and all COVID-19 critical care patients. (PDF)

S3 Table Comparison of characteristics of COVID-19 survivors and deaths. (PDF)

S4 Table Post-infectious and post-acute complications of COVID-19patients transferred out of critical care units. (PDF)

S1 File Post-acute dataset. (XLSX)
