# Supplementary material for: Post-infectious and post-acute sequelae of critically ill adults with COVID-19
Source: PLoS One. 2021 Jun 17;16(6):e0252763. doi: 10.1371/journal.pone.0252763 (PMC8211258; doi:10.1371/journal.pone.0252763)
Supplement: S1 Table — (PDF) [file pone.0252763.s001.pdf]

S1 Table. Characteristics of adult patients transferred from critical care units to general medical ward

	No. (%)		
	All adults (N= 71)	HDU admission (n= 33)	ICU admission (n=38)
**Age, mean, years**			
**<50**	38 (54 %)	15 (45.5%)	23 (60.5%)
**≥50**	33 (46%)	18 (54.5%)	15 (39.5%)
**Sex**			
**Male**	68 (95.8%)	32 (97.0%)	36 (94.7%)
**Female**	3 (4.2%)	1 (3.0%)	2 (5.3%)
**Ethnicity[a]**			
**Arab**	6 (8.4%)	4 (12.1%)	2 (5.3%)
**African**	2 (2.8%)	0 (0%)	2 (5.3%)
**South Asian**	54 (76.1%)	23(69.7%)	31 (81.6%)
**Southeast Asian**	9 (12.7%)	6 (18.2%)	3 (7.9%)
**Comorbidities[b]**			
**No comorbidities**	28 (39.4%)	12 (36.4%)	16 (42.1%)
**Asthma or COPD**	4 (5.6%)	1 (3.0%)	3 (7.9%)
**Hypertension**	14 (19.7%)	8 (24.2%)	6 (15.8%)
**Diabetes**	22 (31.0%)	11 (33.3%)	11 (28.9%)
**Chronic Kidney Disease**	1 (1.4%)	1 (3.0%)	0 (0%)
**Immunosuppression[c]**	2 (2.8%)	1 (3.0%)	1 (2.6%)
**Malignancy[d]**	2 (2.8%)	1 (3.0%)	1 (2.6%)
**Obesity (BMI ≥30)[e]**	18 (25.4%)	11 (33.3%)	7 (18.4%)
**Highest level of respiratory support**			
**Nasal cannula/face mask**	6 (8.4%)	6 (18.2%)	0 (0%)
**High-flow oxygen**	17 (23.9%)	15 (45.5%)	2 (5.3%)
**Noninvasive ventilation**	15 (21.1%)	12 (36.4%)	3 (7.9%)
**Invasive/mechanical ventilation**	33 (46.8%)	0 (0%)	33 (86.8%)

Footnotes Table 1

Abbreviations: HDU, high dependency unit; ICU, intensive care unit; COPD, chronic obstructive pulmonary disease; BMI, body mass index

[a]Race and ethnicity data were collected from UAE national identification cards located in the electronic medical record.

[b]Comorbidities listed here are defined as medical diagnoses included in medical history by ICD-10 coding.

[c]Immunosuppression include HIV, history solid organ transplant or autoimmune disease.

[d]Malignancy includes active solid organ or hematologic malignancy (not in remission) or receiving active chemotherapy.

[e]Obesity was defined as BMI ≥ 30. Body mass index is calculated as weight in kilograms divided by height in meters squared.
