# Supplementary material for: Post-infectious and post-acute sequelae of critically ill adults with COVID-19
Source: PLoS One. 2021 Jun 17;16(6):e0252763. doi: 10.1371/journal.pone.0252763 (PMC8211258; doi:10.1371/journal.pone.0252763)
Supplement: S2 Table — (PDF) [file pone.0252763.s002.pdf]

S2 Table. Demographics of patients transferred from critical care units to general medical ward and all COVID-19 critical care patients

	No. (%)		
	Discharged (N= 71)	All ICU/ HDU admissions (N= 669)	p Value
**Age, mean, years**			
**<50**	38 (54%)	368 (55%)	0.90
**≥50**	33 (46%)	301 (45%)	0.90
**Sex**			
**Male**	68 (95.8%)	589 (88.0%)	**<0.05**
**Female**	3 (4.2%)	80 (12.0%)	**<0.05**
**Comorbidities[a]**			
**No comorbidities**	28 (39.4%)	320 (47.8%)	0.21
**Asthma or COPD**	4 (5.6%)	6 (0.8%)	**<0.05**
**Hypertension**	14 (19.7%)	211 (30.2%)	**<0.05**
**Diabetes**	22 (31.0%)	230 (34.4%)	0.60
**Chronic Kidney Disease**	1 (1.4%)	22 (3.3%)	0.71
**Immunosuppression[b]**	2 (2.8%)	10 (1.5%)	0.32
**Malignancy[c]**	2 (2.8%)	10 (1.5%)	0.32
**Obesity (BMI ≥30)[d]**	18 (25.4%)	170 (25.4%)	1.00

Footnotes Table 2

Abbreviations: HDU, high dependency unit; ICU, intensive care unit; COPD, chronic obstructive pulmonary disease; BMI, body mass index

[a]Comorbidities listed here are defined as medical diagnoses included in medical history by ICD-10 coding.

[b]Immunosuppression include HIV, history solid organ transplant or autoimmune disease.

[c]Malignancy includes active solid organ or hematologic malignancy (not in remission) or receiving active chemotherapy.

[d]Obesity was defined as BMI ≥ 30. Body mass index is calculated as weight in kilograms divided by height in meters squared.
