# Supplementary material for: Post-infectious and post-acute sequelae of critically ill adults with COVID-19
Source: PLoS One. 2021 Jun 17;16(6):e0252763. doi: 10.1371/journal.pone.0252763 (PMC8211258; doi:10.1371/journal.pone.0252763)
Supplement: S3 Table — (PDF) [file pone.0252763.s003.pdf]

S3 Table. Comparison of characteristics of COVID-19 survivors and deaths

	No. (%)		
	Survivor Cohort (N= 71)	Deaths (N= 41)	p Value
**Age, mean, years**			**<0.02**
**<50**	38 (54%)	12 (29.3%)	
**≥50**	33 (46%)	29 (70.7%)	
**Sex**			0.26
**Male**	68 (95.8%)	37 (90.2%)	
**Female**	3 (4.2%)	4 (9.8%)	
**Comorbidities[a]**			
**No comorbidities**	28 (39.4%)	6 (14.6%)	**<0.05**
**Asthma or COPD**	4 (5.6%)	3 (7.3%)	0.71
**Hypertension**	14 (19.7%)	14 (34.1%)	0.11
**Diabetes**	22 (31.0%)	14 (34.1%)	0.83
**Chronic Kidney Disease**	1 (1.4%)	1 (2.4%)	1.00
**Immunosuppression[b]**	2 (2.8%)	0 (0%)	0.53
**Malignancy[c]**	2 (2.8%)	1 (2.4%)	1.00
**Obesity (BMI ≥30)[e]**	18 (25.4%)	12 (29.3%)	0.66

Footnotes Table 3

Abbreviations: HDU, high dependency unit; ICU, intensive care unit; COPD, chronic obstructive pulmonary disease; BMI, body mass index

[a]Comorbidities listed here are defined as medical diagnoses included in medical history by ICD-10 coding.

[b]Immunosuppression include HIV, history solid organ transplant or autoimmune disease.

[c]Malignancy includes active solid organ or hematologic malignancy (not in remission) or receiving active chemotherapy.

[d]Obesity was defined as BMI ≥ 30. Body mass index is calculated as weight in kilograms divided by

height in meters squared.
