# Supplementary material for: Post-infectious and post-acute sequelae of critically ill adults with COVID-19
Source: PLoS One. 2021 Jun 17;16(6):e0252763. doi: 10.1371/journal.pone.0252763 (PMC8211258; doi:10.1371/journal.pone.0252763)
Supplement: S4 Table — (PDF) [file pone.0252763.s004.pdf]

S4 Table. Post-infectious and post-acute complications of COVID-19 patients transferred out of critical care units

Clinical Outcome	No. (%)<br>All adults<br>(N= 71)	HDU admission<br>(n= 33)	ICU admission<br>(n= 38)
**Length of stay[a], mean, days (SD)**	12.4 (5.29)	13.4 (4.53)	12.3 (5.29)
**Renal**	68 (95.8%)		
Electrolytes Imbalance	49 (69.0%)	14 (42.4%)	35 (92.1%)
Acute Kidney Injury[b]	11 (15.5%)	2 (6.1%)	9 (23.7%)
Renal Replacement Therapy	8 (11.3%)	0 (0.0%)	8 (21.1%)
**Hepatic**			
Acute Hepatic Injury[c]	11 (15.5%)	6 (18.2%)	5 (13.2%)
**Cardiac**			
Acute Cardiac Injury[d]	1 (1.4%)	0 (0.0%)	1 (2.6%)
**Pulmonary**			
Post-acute supplemental oxygen	48 (67.6%)	22 (66.7%)	26 (68.4%)
**Hematologic**			
VTE	1 (1.4%)	0 (0.0%)	1 (2.6%)
**Musculoskeletal**	44 (62.0%)		
Myopathy[e]/muscle weakness	22 (31.0%)	1 (3.0%)	21 (55.3%)
Pressure ulcer/wound	22 (31.0%)	1 (3.0%)	21 (55.3%)
**Neurologic**	39 (54.9%)		
Delirium/confusion[f]	13 (18.3%)	0 (0.0%)	13 (34.2%)
Ataxia/gait imbalance	4 (5.6%)	0 (0.0%)	4 (10.5%)
Swallowing impairment	22 (31.0%)	1 (3.0%)	21 (55.3%)

Footnotes Table 4

Abbreviations: HDU, high dependency unit; ICU, intensive care unit; SD, standard deviation; VTE, venous thromboembolism

[a]Length of stay begins with admission time and ends with discharge time, days

[b]Acute kidney injury was identified as an increase in serum creatinine by ≥0.3mg/dL (≥26.5 mol/L) within 48 hours or an increase in serum creatinine to ≥1.5 times baseline within the prior 7 days compared with the preceding 1 year of data in acute care medical records. Acute kidney injury is calculated only for patients with record of baseline kidney function data available and without a

diagnosis of end-stage kidney disease.
